# Adding a Brief Continuous Glucose Monitoring Intervention to the National Diabetes Prevention Program: A Multimethod Feasibility Study

**DOI:** 10.1155/2024/7687694

**Published:** 2024-05-16

**Authors:** Kelli M. Richardson, Susan M. Schembre, Vanessa da Silva, Robert M. Blew, Nick Behrens, Denise J. Roe, Farshad Fani Marvasti, Melanie Hingle

**Affiliations:** ^1^School of Nutritional Sciences and Wellness, College of Agriculture, Life and Environmental Sciences, University of Arizona, Tucson, Arizona, USA; ^2^Department of Oncology, Lombardi Comprehensive Cancer Center, Georgetown University, Washington, DC, USA; ^3^Department of Ecology and Evolutionary Biology, College of Science, University of Arizona, Tucson, Arizona, USA; ^4^Department of Epidemiology and Biostatistics, Mel and Enid Zuckerman College of Public Health, University of Arizona, Tucson, Arizona, USA; ^5^Department of Family, Community and Preventive Medicine, College of Medicine, University of Arizona, Phoenix, Arizona, USA

## Abstract

The National Diabetes Prevention Program (DPP) promotes lifestyle changes to prevent diabetes. However, only one-third of DPP participants achieve weight loss goals, and changes in diet are limited. Continuous glucose monitoring (CGM) has shown potential to raise awareness about the effects of diet and activity on glucose among people with diabetes, yet the feasibility of including CGM in behavioral interventions for people with prediabetes has not been explored. This study assessed the feasibility of adding a brief CGM intervention to the Arizona Cooperative Extension National DPP. Extension DPP participants were invited to participate in a single CGM-based education session and subsequent 10-day CGM wear period, during which participants reflected on diet and physical activity behaviors occurring prior to and after hyperglycemic events. Following the intervention, participants completed a CGM acceptability survey and participated in a focus group reflecting on facilitators and barriers to CGM use and its utility as a behavior change tool. A priori feasibility benchmarks included opt-in participation rates ≥ 50%, education session attendance ≥ 80%, acceptability scores ≥ 80%, and greater advantages than disadvantages of CGM emerging from focus groups, as analyzed using the Key Point Summary (KPS) method. Thirty-five DPP members were invited to participate; 27 (77%) consented, and 24 of 27 (89%) attended the brief CGM education session. Median survey scores indicated high acceptability of CGM (median = 5, range = 1–5), with nearly all (*n* = 23/24, 96%) participants believing that CGM should be offered as part of the DPP. In focus groups, participants described how CGM helped them make behavior changes to improve their glucose (e.g., reduced portion sizes, increased activity around eating events, and meditation). In conclusion, adding a single CGM-based education session and 10-day CGM wear to the DPP was feasible and acceptable. Future research will establish the efficacy of adding CGM to the DPP on participant health outcomes and behaviors.

## 1. Introduction

More than one-third of US adults have prediabetes [1], and up to 70% of individuals with prediabetes progress to type 2 diabetes (T2DM) [2], a serious metabolic disease conferring increased risk of cardiovascular disease and stroke [3], and contributing to greater lifetime disability, economic burden [4], and premature death [3, 5]. Prediabetes can be managed and even reversed with lifestyle changes, including dietary modification and regular physical activity [6].

The efficacy of lifestyle behavior change interventions for diabetes prevention is well established. Findings from the US Diabetes Prevention Program (DPP), a randomized clinical trial assessing the effects of a lifestyle intervention program on body weight and physical activity among > 3,000 adults with prediabetes, demonstrated a 58% reduction in the risk of incident T2DM [7], with 10- and 15-year follow-up data confirming sustained effects, including significant reductions in diabetes progression [8, 9]. Similar follow-up data from two diabetes prevention trials conducted in China and Finland showed similar reductions in risk of progression to T2DM over time (39% reduction at 30 years [10] and 43% reduction at 7 years [11], respectively). These findings have collectively informed the US National DPP, a 12-month Centers for Disease Control and Prevention (CDC) program designed to support participants in modifying lifestyle behaviors (physical activity, diet) to promote weight loss. The National DPP is intended for adults ≥ 18 years old with overweight or obesity who are at high risk for developing T2DM and is offered via in-person, distance learning (remote, synchronous), online (self-paced, asynchronous), or a combination of modalities, all of which have been shown to produce similar National DPP outcomes [12]. A recent study of National DPP participants (*N* = 14,737 across 220 US-based programs) showed that only one-third of participants met program weight loss goals [13]. Cited barriers to program attendance and completion, including scheduling and transportation difficulties, lack of support from family and friends, low confidence, and lack of resources to support recommended changes [14], contribute to less than optimal outcomes. Additionally, recent evidence suggests that the National DPP does not promote meaningful changes in measures of diet quality relevant to diabetes risk [15]. Therefore, revisions to the National DPP curriculum that include educational tools to help participants achieve dietary goals and improve diabetes prevention outcomes may be warranted [15].

Recent advances in wearable biosensors and the ubiquity of connected devices provide novel and engaging opportunities to enhance National DPP prevention efforts through personalized and continuous biological feedback [16, 17]. Biological feedback has primarily been used as a behavior change technique in diabetes research with recent studies using continuous glucose monitors (CGM) to collect glucose and glucose trend data to provide to the user via a connected device [18]. Allowing participants to regularly monitor their glucose data can offer them valuable insights into how their diet and physical activity influence their glucose levels which, in turn, may motivate them to adjust these behaviors to enhance their diabetes prevention efforts. Several systematic reviews and meta-analyses have shown that people with T2DM, who use CGM, have greater reductions in glycated hemoglobin (HbA1c) compared to those using traditional blood glucose self-monitoring methods [19, 20], and when combined with education and personalized lifestyle behavior change plans as part of a T2DM care program, glycemic control was further optimized [21].

CGMs are now available to be prescribed at the discretion of one's healthcare provider and can include reimbursement for populations without diabetes [22]. This may explain the recent emergence of CGM-based clinical trials for people without diabetes [23, 24]. To date, however, there has been little behavioral intervention research conducted in people with prediabetes using CGM [25]. The objective of this study was to assess the feasibility of a brief intervention consisting of a single 60-min CGM-based education session followed by 10 days of CGM wear conducted within the context of the Arizona Cooperative Extension National DPP.

## 2. Materials and Methods

This study follows the Consolidated Standards of Reporting Trials (CONSORT) extension for Pilot and Feasibility Trials [26]. The study was approved by the University of Arizona Institutional Review Board (STUDY00001809).

### 2.1. Recruitment and Eligibility

Participants from English-speaking, distance-mode Arizona Cooperative Extension National DPP cohorts were invited to opt-in to receiving a brief CGM-based intervention in addition to the original program sessions. The number of National DPP cohorts approached was dependent upon opt-in rate; the recruitment goal of 30 participants was achieved after two cohorts were approached. A graduate research associate attended a National DPP session held over Zoom 1 month prior to the study start date and verbally described the study and methods, then provided National DPP participants with a secure web link containing an interest form and an eligibility screening form. Eligible participants met the CDC National DPP eligibility criteria (≥ 18 years old, BMI ≥ 25 or ≥ 23 if Asian, no previous diagnosis of T1DM or T2DM, and at high risk of diabetes as determined by a clinical diagnosis of prediabetes; or a history of gestational diabetes; or a high score on the American Diabetes Association T2DM risk assessment questionnaire) and were willing to wear a CGM. Interested and eligible participants were instructed to download the MyDataHelps (CareEvolution, LLC) app—a digital clinical trial and research platform that is secure for HIPAA-regulated data—to their personal mobile device which they used to complete the informed consent document.

### 2.2. Intervention Procedures

To align with the delivery mode of most postpandemic Arizona Cooperative Extension National DPP cohorts, this intervention was remotely delivered. Two weeks prior to the intervention, participants received a mailed package containing instructions, two Dexcom G6 CGM devices (Dexcom, San Diego, CA, USA), an ActiGraph GT9X Link accelerometer (ActiGraph, Pensacola, FL, USA), a Fitbit Charge 5 health and fitness tracker (Fitbit Inc., San Francisco, CA, USA), and an A1cNow Self Check portable HbA1c detection kit (PTS Diagnostics, Whitestown, IN, USA). Starting at week 0, participants were instructed to wear a blinded CGM on their abdomen for 10 days to become acquainted with the sensor. During this 10-day period, participants also wore the accelerometer on their hip, completed an A1c test, and logged their weight and self-reported physical activity using the MyDataHelps app. Participants also completed a questionnaire containing a 4-item Healthy Days Core Module (CDC HRQOL-4) [27] to assess quality of life and two questions to assess self-efficacy to change diet and activity. Two, 24-h dietary recalls guided by trained interviewers using the USDA multiple-pass method were also administered during the 10-day period [28]. The accelerometer, A1c test, questionnaires, and dietary recalls were repeated at week 12.

During week 2, participants received one, 60-min education session led by a trained research assistant using Zoom for Health, a digital, HIPAA-compliant conferencing platform. The session oriented participants to the sensor and described ways in which diet and physical activity could affect their glucose levels. Participants also learned how to make diet and activity modifications to limit glucose excursions above 140 mg/dL, [29] and how they could use CGM data to identify foods more likely to elevate their glucose levels. Participants were encouraged to use the MyDataHelps app to track what they ate, the time they ate, their highest peak postprandial glucose level, and any changes that they planned to make to limit a glucose excursion after a similar meal. Participants also received a list of commonly consumed high glycemic foods, which was sourced from food records collected as part of a prior study conducted in residents of Southern Arizona with similar demographics. At the conclusion of the CGM education session, participants were instructed to self-apply a second, unblinded CGM following manufacturer user guidelines. One participant contacted the study team for additional assistance; all others successfully self-applied their CGM. Participants wore the unblinded CGM for up to 10 days along with a wrist-worn Fitbit Charge 5. Median wear time was 10 days (range: 9–12 days), and median data sufficiency was 100% (range: 98–100%).

### 2.3. Quantitative Methods

The primary outcomes were feasibility and acceptability. Feasibility was assessed by demand and practicality per Bowen et al.'s criteria [30]. Demand was measured by the number of National DPP members who expressed written interest to participate compared to the total number of National DPP members in the cohort(s). Practicality was measured by the proportion of enrolled participants who attended the CGM education session.

Acceptability was measured using a 10-item survey consisting of 5-point Likert scale questions used in previously published CGM-based work where scores of 5 were most favorable [31]. Additional questions regarding the acceptability of the CGM education session and the duration of CGM wear were added. The survey was delivered during week 4, immediately after the 10-day unblinded CGM wear period, through the MyDataHelps app.

Study opt-in rates were computed as the percent of total cohort participants who expressed written interest to participate in the study. Descriptive statistics were summarized for the survey data. Because this was a feasibility study, we were not statistically powered to analyze the 3-month pre- and postintervention data; therefore, they are not presented here.

### 2.4. Qualitative Methods

During week 4, participants were invited to join a virtual focus group (Zoom for Health). The goal of the focus group was to elicit participant feedback regarding intervention feasibility and acceptability, including facilitators and barriers to intervention participation and perceived fit with the Arizona Cooperative Extension National DPP curriculum and program scheduling. The degree to which participants used CGM data to make diet and physical activity changes was explored. Participants were also invited to share any challenges experienced in taking part in the intervention or completing surveys or measurements. Focus groups (a total of four to accommodate participant schedules) were led by a trained research assistant using a semistructured script (Supporting Information available here). Focus group discussions were digitally recorded and transcribed (GMR Transcription). The Key Point Summary (KPS) method was used to analyze qualitative data gathered during and after each focus group [32]. This method involved a rapid summary of the major points from the focus groups and salient respondent quotes. A single research assistant developed a codebook and independently coded each of the four focus groups using Dedoose (v9.0.90). Based on coded excerpts, a single KPS for all focus groups was developed.

### 2.5. Measures

A priori benchmarks were set by the research team. The brief CGM intervention was deemed feasible if ≥ 50% of invited National DPP members opted-in to the study, and ≥ 80% of enrolled participants attended the CGM education session. Intervention acceptability was indicated if median survey scores were ≥ 80% (≥ 4 on a 5-point Likert scale), and if focus group discussions revealed more facilitators than barriers of using CGM to prompt health behavior changes.

## 3. Results

### 3.1. Participants

**Figure 1:**
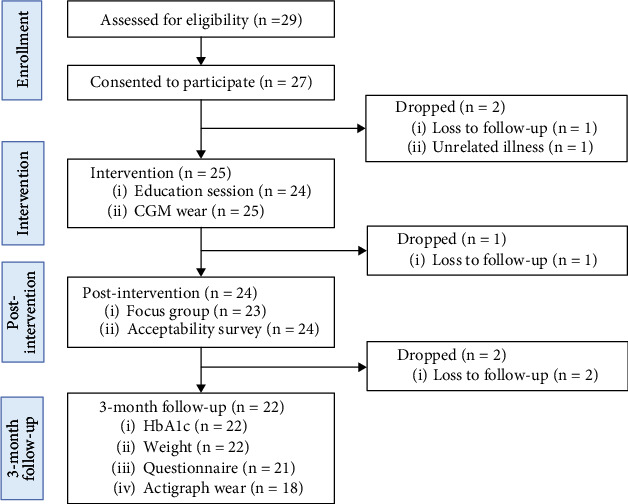
CONSORT diagram.

Figure 1 depicts the flow of participants from recruitment through the end of the study. We invited participants from two National DPP cohorts, consisting of 35 total participants, to join the study. Twenty-nine of 35 (83%) National DPP participants expressed interest and completed the eligibility screening questionnaire. All twenty-nine respondents were eligible, and *n* = 27 (77%) provided written consent to participate and were enrolled in the study. Two of 27 (7%) participants dropped out of the study prior to receiving the brief CGM intervention. One of the remaining 25 participants (4%) completed the intervention but did not complete the postintervention survey or focus group. Of the remaining 24 participants, *n* = 2 (8%) were lost to follow-up at 3 months.

Table 1 describes the characteristics of the *N* = 27 enrolled participants. A majority of participants were female (*n* = 26, 96.3%), White (n =21, 77.8%), and highly educated (college graduate; *n* = 17, 63.0%) with a high household income (≥$75,000 annually; *n* = 12, 44.4%). Their average age was 54 years (SD = 13.24).

### 3.2. Quantitative Data

#### 3.2.1. Feasibility

Demand to participate exceeded our a priori criteria of a 50% opt-in rate. Twenty-seven individuals enrolled in the study, and *n* = 24 (89%) attended the CGM education session. This attendance rate surpassed our a priori criteria of ≥ 80%, confirming the overall feasibility of the intervention.

#### 3.2.2. Acceptability

Participants rated all aspects of the CGM education session highly. On a 5-point successive Likert scale survey (where 1 = *strongly disagree* and 5 = *strongly agree*), participants found the information presented in the session to be relevant (median = 4; range = 4–5). Participants agreed that CGM helped them to better understand the relationship between their diet and glucose (median = 5; range = 2–5) and physical activity and glucose (median = 5; range = 2–5). A majority of participants agreed that after the education session, using CGM increased their motivation to make dietary changes (median = 5; range = 3–5) and be more physically active (median = 5; range = 2–5).

Table 2 presents the median and range for CGM acceptability survey questions. Twenty-four of 27 enrolled participants (89%) completed the postintervention acceptability survey. Participants rated CGM as highly acceptable, with an overall average score of 4.50 ± 0.74 (90%), which exceeded our a priori criteria of ≥ 4 out of 5 (≥ 80%). In addition, scores indicated that CGM provided information that was of interest to participants (median = 5; range = 4–5), and that CGM was useful and beneficial (median = 5; range = 4–5).

Related to CGM usage and wear duration, *n* = 21/24 (88%) participants stated that they would wear the CGM again if given the opportunity. A majority of participants (*n* = 15/24, 63%) indicated that they would be willing to wear CGM for more than 3 months in a row, and a majority (*n* = 17/24, 71%) also stated that they would wear CGM every month if provided the opportunity. An overwhelming majority (*n* = 23/24, 96%) indicated that they believed CGM should be offered regularly as part of the National DPP.

### 3.3. Qualitative Data

Twenty-three of 27 participants (85%) participated in the focus group. Key points emerging from the discussion included advantages and disadvantages of using CGM, the impacts of behavior (specifically diet, activity, and stress management) on their glucose levels, barriers to behavior change, and considerations for a future program.

#### 3.3.1. Advantages and Disadvantages of CGM

Most participants mentioned advantages of CGM compared to disadvantages. In contrast to the traditional blood glucose monitor, participants enjoyed how the CGM did not require finger pricks, finding the sensor to be noninvasive and comfortable. Participants also appreciated being able to see the immediate impact of their behaviors on their glucose levels (as compared to the longer duration to see the impact of behaviors on body weight) and make changes to food and activity choices in response to their glucose data. Participants described how using CGM and seeing their data encouraged them to make healthy decisions and made them realize that they have control over their glucose levels. Several participants also commented on their ability to experiment with various foods and activities to find the combination that works best for their personal glucose levels. Participants also used CGM as an educational tool for others, showing their friends and family the impact food has on glucose levels. Overall, participants described CGM as being motivational, reassuring, comforting, and helpful in holding them accountable.

There were several technical issues mentioned related to the Dexcom G6 app and the device itself, with the most frequently cited issue being the placement of the CGM on the abdomen. Participants noted that it would often get caught on their pants or undergarments and that they would have preferred an arm placement. Some participants experienced irritation and itchiness while wearing the CGM, and one participant experienced bruising after taking it off. One participant disliked how she could not remove it during the 10-day wear period, so she could not clean that area of her skin. Several participants expressed frustration while using the Dexcom G6 app, with the main concern being that they lost Bluetooth connection. Participants mentioned how they do not typically keep their phone near them and suggested providing a pouch (e.g., lanyard) to carry their phone in. Other app-related issues included difficulty viewing their glucose data (including the numerical value associated with their glucose peak and historical CGM data), and issues with smartphone compatibility with the Dexcom G6 app, suggesting that additional support might be needed to help participants optimize their user experience. One participant expressed concerns about the difference between her CGM- and glucometer-derived data.

Additionally, the concept of CGM-related distress emerged from the focus groups as a disadvantage of CGM. A few participants experienced frustration while wearing CGM, with one describing how “everything” caused her glucose levels to rise, while another participant described how her glucose levels would begin to rise, but she was too fatigued to exercise again. One participant stated that CGM caused her to be discouraged when she would attempt physical activity to reduce her glucose levels, but her glucose levels would remain elevated. Others described being upset and worried at seeing their high glucose levels because it served as a constant reminder of their prediabetes status. One participant described her CGM wear experience as an “emotional rollercoaster,” as she thought she was eating “good,” but her CGM would show otherwise. Others expressed how they became obsessive in viewing their glucose levels, with one participant stating that it was frightening how often she looked at her numbers, and another participant stating she experienced “compulsive” behaviors related to checking her numbers. A few participants also noted that the CGM alarms scared them and caused anxiety. Lastly, one participant's distress stemmed from the device itself, as she described her experience of putting the CGM on and taking it off as “scary” and “nerve-racking.”

#### 3.3.2. Impact of Behavior on Glucose Levels

Participants described using the CGM to learn about the relationship between their diet, physical activity, and stress management behaviors and their glucose levels.

##### 3.3.2.1. Diet

As a result of using CGM, the most-cited dietary behavior change was consuming more balanced meals and snacks (i.e., incorporating proteins and fats into carbohydrate-containing dishes). One participant noted that this would make her glucose levels stay higher for longer, but her glucose would not rise as high as compared to when a carbohydrate-only dish was consumed. Another commonly mentioned dietary behavior change was reducing portion sizes. Several others mentioned making changes to specific foods, such as reducing the amount of dressing used on salads, replacing flour tortillas with corn tortillas, removing the oat topping from yogurt, and eliminating sugary beverages. Many participants reflected on how they were surprised by their glucose levels after eating certain foods that they had thought would not result in a glucose excursion. Some of these foods included oatmeal, whole wheat bagel with cream cheese, bran cereal, potatoes, cauliflower crust, and small portions of fudge or a small cookie. On the contrary, participants noted that both fruits and vegetables did not cause their glucose levels to rise as high, prompting them to include more fruits and vegetables in their diet. Several participants mentioned experimenting with foods so that they could use the information gained to formulate better eating habits. Others focused on the time of day that caused glucose levels to rise, with one participant describing how her glucose levels were highest after morning meals, so she had been putting a greater emphasis on adjusting her breakfast foods (as compared to other meals). Lastly, participants reflected on their experience, noting that it brought about a greater awareness about the impact of food on glucose levels. For a few participants, CGM made them reflect on how they ate their entire lives, motivating them to change their dietary behaviors moving forward.

##### 3.3.2.2. Activity

Participants also acknowledged the impact of physical activity on their glucose levels. One participant stated the following: “Exercise really did produce a change for me. And I think that was kind of cool because I didn't know that before going into the study that it would help that much.” Several participants noted the effect of exercise on glucose levels, recognizing that if their glucose levels did rise, there was something that they could do about it. Two behavior changes mentioned by most participants were increasing overall activity and the timing of activity around glucose excursions. One participant mentioned that she would initiate activity after seeing her glucose rise, even if she had already completed her recommended amount of activity. Participants noted that strenuous activity was not needed to reduce glucose levels—walking down the hallway at work, around the island of their kitchen, at a shopping mall, or even in place helped to reduce their glucose levels. Participants also noted that the type of activity made a difference—walking and dancing seemed to help, while biking did not help. Lastly, one participant mentioned how instead of being active for one bout of 30 min, she was active for multiple bouts of 10 min around eating events.

##### 3.3.2.3. Stress Management

Although psychological stress was not discussed in the CGM education session provided as part of the intervention, several participants noted the impact of stress on their glucose levels. One participant described how on days where she felt more stressed, she noticed higher glucose levels, prompting her to make stress management a priority. Another noted the positive impact of breathing exercises and meditation on her glucose levels. One participant shared how she experienced a stressful family event and immediately looked at her CGM to find that her glucose levels had significantly increased.

#### 3.3.3. Barriers to Behavior Change

Despite the observed benefits of dietary and physical activity changes, there were several barriers to implementing changes throughout the CGM wear period. Participants mentioned how they would change their diet to incorporate foods that would limit their glucose excursions; however, this would also result in them not feeling full and snacking later. A few participants noted this specifically with breakfast foods; they would not feel full after eating eggs, but other “typical” breakfast foods would cause their glucose levels to rise. Some participants also noted the difficulty in finding time to plan and prepare meals that would both keep them full and limit their glucose excursions. Several participants mentioned how their environments made it difficult to choose foods that would limit glucose excursions, such as partners eating different foods than them, neighbors bringing them baked goods, supporting Girl Scouts by purchasing cookies, and eating with others during the holidays. One participant noted difficulty with selecting foods that would not cause a glucose excursion when she had cravings. In terms of activity, participants stated that it was difficult to be active in general (independent of CGM). One participant spoke of one evening when she saw her glucose levels rise, but she had felt too fatigued to be active. Others were discouraged when they saw no impact of physical activity on their glucose levels. Lastly, one participant was cautious about being physically active as she was concerned about bending over and stretching with the CGM on her abdomen.

#### 3.3.4. Considerations for Future Programs

When discussing considerations for a future program that incorporates CGM into the National DPP, participants discussed (1) their preferred duration of CGM wear, (2) intervention logistics, and (3) additional resources they desired.

##### 3.3.4.1. Duration of CGM Wear

All participants expressed an interest in wearing the CGM more than one time. Several participants commented on how they used the 10-day wear period to experiment with different foods and activities, but there are more experiments that need to be done to discover which diet and exercises (including the type, duration, pace, and timing) work best for them. Some had the expectation that they could be “perfect” by the end of the 10-day wear period but admitted that 10 days were not enough time. A few participants also mentioned that the timing was poor (e.g., a “bad week” for them, being sick) and wished that they could try the CGM again during a better week.

Most participants wished that they could wear a CGM all of the time. Others suggested time periods ranging from one more month to three more months. Some wished that it could be made available throughout the entire duration of the National DPP (12 months) despite whether they chose to wear it the entire duration or not. One participant suggested having one CGM at week 8 of the National DPP, another CGM halfway through the National DPP, and one at the end of the National DPP to view progress. It was noted by two participants that starting CGM after seven or eight sessions was appropriate, as compared to having it on the 1st day of the National DPP.

##### 3.3.4.2. Intervention

Participants provided feedback on the 1-h CGM education session and glucose tracking homework. Overall, participants thought that the education session was well-explained, and they appreciated the ample time provided for questions. In particular, participants noted that they liked the recommendation of being physically active for 30–45 min either before or after eating, as provided in the education session. One participant commented on the long duration (2 h) that the CGM takes to initially sync with the app and wished that she could have seen the data earlier so that she knew what questions to ask during the session. Two suggestions of topics to cover during the education session were potential consequences of glucose levels > 140 mg/dL and an emphasis on keeping the phone nearby to avoid Bluetooth disconnection issues. Lastly, one participant stated that it was slightly redundant of what was covered previously in the National DPP, and another participant stated that some of the foods mentioned in the CGM education session did not align with what was recommended in previous National DPP sessions.

Several participants noted the benefit of reflecting on why their glucose levels rose above 140 mg/dL, stating that it made them think about what they could do differently in the future. Some participants mentioned that they were unsure why a particular meal caused their glucose levels to rise. A few participants noted the complexity of reporting multi-ingredient dishes (e.g., homemade vegetable soup) as part of the reflection. One participant experienced confusion when reporting their dietary intake in the MyDataHelps app. Another participant disliked the use of multiple apps (MyDataHelps, Dexcom G6, Fitbit) for tracking and reflection. Lastly, there were a few participants who stated that simply in general, it is difficult to build the habit of tracking foods and times eaten.

##### 3.3.4.3. Additional Resources Desired

A variety of additional resources were suggested to improve the CGM intervention. The most frequently mentioned suggestion was the ability to work one-on-one with a dietitian to review their CGM data and to receive individualized recommendations based on foods that did or did not spike their glucose levels. Specifically, participants wanted to know what they did well and how they could improve. Conversely, two participants suggested receiving more general education on “good” versus “bad” foods, as opposed to personalized recommendations. Some participants additionally requested a summary report so that they can view their overall trends (as opposed to their real-time daily data) and share their reports with their doctors.

Multiple participants suggested a more intense intervention. Several recommended having more in-depth journaling so that they could use these notes beyond the CGM wear period. One participant also recommended having a notification be sent each time their glucose rises above 140 mg/dL as a reminder.

Participants also desired additional education on what (besides food and activity) can cause glucose to fluctuate. Participants suggested having a past participant (or user of CGM) visit to give advice on topics such as these that would not otherwise be listed on directions and to share their success stories of using CGM. Some participants were also curious about the difference between glucometer and CGM readings and expressed the desire to check. Several participants also requested additional resources on how to obtain a CGM outside the study, with specific concerns about insurance coverage. Several participants also mentioned family members or friends with T2DM and inquired about resources for them as well.

Lastly, multiple participants commented on their appreciation of the support line provided by the research assistant via either email, text, or phone call. They stated that it was easy to ask for help and that response times were fast. Some participants recognized that while they did not use the support line, they felt comfort knowing someone was there if needed.

## 4. Discussion

To our knowledge, this was the first study to assess the feasibility and acceptability of a brief CGM intervention added to the National DPP, and among the first to investigate the use of CGM as a behavior change tool for people with prediabetes. Based on our quantitative data relative to a priori criteria and predominantly positive qualitative data, the CGM intervention was deemed both feasible and acceptable. A majority (*n* = 27/35, 77%) of National DPP members who we attempted to recruit consented to participate, demonstrating initial attraction to using CGM in this population. After using the device, participants expressed high acceptability, with nearly all participants (*n* = 23/24, 96%) believing that CGM should be offered regularly as part of the National DPP.

Participants rated CGM highly using the same survey as Liao and Schembre who observed similar results in their CGM intervention for persons with overweight and obesity (*N* = 19), with an average CGM acceptability rating of 4.46 out of 5 [33]. A majority of participants in the current study (*n* = 21/24, 88%) expressed interest in using CGM again; similar interest was demonstrated in a study by Schembre et al., wherein 87% (*n* = 13/15) of postmenopausal women without diabetes were also interested in wearing a CGM again [34]. Overall, the preliminary acceptability findings from the present trial and in previous literature support the use of CGM in populations without diabetes.

Participants reported using CGM to guide dietary changes, including incorporating protein and fats in carbohydrate-containing meals. Similar changes were made by participants with prediabetes in a study by Yost et al., where participants were able to notice the impact of carbohydrates on their glucose levels, driving changes in their dietary patterns [35]. Additionally, participants in the current study used CGM to determine which foods they would discontinue eating, including eliminating sugary beverages and oats on top of their yogurt. Whelan et al. demonstrated similar findings, with participants stating that the CGM provided physiological evidence of the effects of certain foods on their glucose levels, which made eliminating foods from their diet easier [36]. Participants reported enjoying food experimentation, which also occurred in other studies [36]. Because of this, interpreting changes in diet and glycemic control while wearing CGM should be done with caution. While participants may be making positive changes to their diet while wearing CGM, they may also be experimenting with lower nutrient quality foods, which could negatively affect CGM and dietary data, inaccurately capturing dietary changes. Despite this limitation, the present study and previous literature suggest that CGM may be used as a tool to support dietary changes in people with prediabetes.

Finally, participants expressed that CGM motivated them to increase their physical activity. These findings are supported by Liao and Schembre, where participants transitioned from the precontemplation stage of behavior change to the action stage after just one physical activity-focused education session followed by 10 days of CGM wear [33]. Participants in the current study indicated that CGM helped them to adjust the timing of their activity, timing their activity bouts with eating events to minimize postprandial glucose excursions. Whelan et al. demonstrated similar findings—participants at risk for T2DM using CGM discovered that being active, even just for a short period of time such as taking their dog outside for 10 min, brought down their glucose levels [36]. On the other hand, some participants in the current study reported seeing no changes in their glucose levels following physical activity. Similar findings were shown in the study by Whelan et al., with some participants stating that they could not see a clear relationship between activity and glucose [36]. There may be several explanations for this. A study of 5157 participants with diabetes demonstrated that while a majority (76%) of participants experienced reduced glucose levels after activity, the remaining 24% either had increased or unchanged glucose levels following activity, demonstrating that some individuals may not respond favorably to activity [37]. Another explanation for this may be that higher intensity activity results in increased stress hormones (e.g., adrenaline) which can increase glucose levels [38]. Furthermore, some participants in the current study expressed that while some activities such as walking do produce a change, other activities such as biking do not. While Colberg, Hernandez, and Shahzad showed that cycling reduces glucose levels more than walking in people with diabetes, the null response to biking experienced by participants in the current study may be due to variations in intensity, duration, or timing of exercise, all of which can produce varying effects on an individual's glucose response [37].

Collectively, these findings can be utilized to refine future CGM-based intervention work. Future research will address the advantages and disadvantages identified by focus group participants, with the intent of improving the efficacy of this approach while continuing to optimize acceptability.

### 4.1. Strengths and Limitations

There were several strengths and limitations of this study. Our collaboration with the Arizona Cooperative Extension National DPP allowed us to recruit participants with prediabetes, an understudied population in general (given that the majority of people in the United States that have prediabetes do not know it) [39] and specifically with regard to CGM studies [25]. Additionally, the Arizona Cooperative Extension National DPP State Director and several lifestyle coaches reviewed our education session materials and provided feedback to ensure that our materials were consistent with other National DPP sessions and to remain closely aligned with the National DPP, further facilitating integration with the program. We used both quantitative and qualitative measures, which allowed us to gain deeper insights into the experience of participants. We set a priori criteria to assess both feasibility and acceptability, limiting the potential for reporting bias.

Despite these strengths, there were also several limitations. Our sample was composed of mostly white, educated females. While this may limit the generalizability of these results for people with prediabetes, the population is representative of those enrolled in the National DPP, which was our primary recruitment goal. Another potential limitation is the simultaneous wear of CGM and Fitbit. This additional device limits our ability to discern whether changes to physical activity, as mentioned through focus groups, were attributable to the biological feedback from the CGM or Fitbit. We did not collect dietary intake data during the 10-day CGM intervention; therefore, self-reported changes in diet as described in the focus group were not supported by quantitative data. Another potential limitation is that the qualitative data was coded by one research assistant, which may limit reliability. To address this limitation, results of the qualitative assessment were reviewed and accepted by study PIs. Additionally, this study was remotely conducted; future research should investigate in-person and distance learning modalities. In anticipation of this future work, our intervention protocol was designed to be delivered across different modalities, similar to the National DPP [12]. Finally, for one cohort, the brief intervention was delivered during week 9 of the National DPP which was in December; wearing a CGM during the December holidays may have captured atypical behaviors. A future definitive clinical trial will address these limitations and test the efficacy of our intervention on behavioral and biological outcomes.

## 5. Conclusions

Adding a brief CGM intervention, consisting of one CGM-based education session followed by 10 days of CGM wear, to the Arizona Cooperative Extension National DPP was feasible and acceptable. Qualitative findings suggested that participants used CGM to inform dietary, activity, and stress management behavior changes. Additional research is necessary to determine the efficacy and cost-effectiveness of this intervention on biological and behavioral outcomes.

## Figures and Tables

**Table 1:** Participant characteristics (*N* = 27).

**Characteristics**	**Values**
Age, mean (SD)	54.15 (13.24)
Sex, *n* (%)	
Male	1 (3.7%)
Female	26 (96.3%)
Nonbinary	0 (0.0%)
Race, *n* (%)	
American Indian or Alaska Native	1 (3.7%)
Asian	0 (0.0%)
Black or African American	3 (11.1%)
Native Hawaiian or Other Pacific Islander	0 (0.0%)
White	21 (77.8%)
More than one race	2 (7.4%)
Ethnicity, *n* (%)	
Hispanic or Latino	6 (22.2%)
Not Hispanic or Latino	21 (77.8%)
Highest level of education, *n* (%)	
Less than grade 12	0 (0.0%)
Grade 12 or GED	3 (11.1%)
College: 1 to 3 years (some college or technical school)	7 (25.9%)
College graduate (4 years or more)	17 (63.0%)
Income, *n* (%)	
$0–24,999	2 (7.4%)
$25,000–$49,999	5 (18.5%)
$50,000–$74,999	7 (25.9%)
Greater than $75,000	12 (44.4%)
Decline to answer	1 (3.7%)
HbA1c (*N* = 26)	
Median (range)	5.8 (5.2–6.4)
Average (SD)	5.8 (0.3)
Percent time in range (70–180 mg/dL; *N* = 25)	
Median (range)	98.7 (69.0–100)
Average (SD)	96.6 (6.5)

**Table 2:** Participants' acceptability of using CGM based on a 5-point Likert scale survey (*n* = 24).

**Survey item**	**Median ** ^**a**^	**Range**
Relevance: CGM provides information that is of interest to me.	5	4–5
Value: CGM is useful and beneficial.	5	4–5
Usability: CGM is easy to use and user-friendly.	5	3–5
Recommend: I would recommend CGM to my friends and family.	5	3–5
Convenience: CGM is convenient for me to use in my everyday life.	5	3–5
Tech support: There is adequate availability and quality of professional assistance throughout the use of the CGM.	5	3–5
Motivating: I am motivated to use CGM to track my daily behaviors.	5	2–5
Like: I like using the CGM.	5	1–5
Confidence: I feel confident that I use CGM correctly.	4.5	3–5
Privacy: I am concerned about my privacy when using CGM.	2	1–4

^a^The Likert scale scores were 1 = *strongly disagree*, 2 = *disagree*, 3 = *neither agree nor disagree*, 4 = *agree*, and 5 = *strongly agree*.

## Data Availability

The raw data used to support the findings of this study may be available from the corresponding author upon request.
