# Supplementary material for: Adding a Brief Continuous Glucose Monitoring Intervention to the National Diabetes Prevention Program: A Multimethod Feasibility Study
Source: J Diabetes Res. 2024 May 16;2024:7687694. doi: 10.1155/2024/7687694 (PMC11199067; doi:10.1155/2024/7687694)
Supplement: Supporting Information — Additional supporting information can be found online in the Supporting Information section. Supporting information include the semistructured focus group guide. [file 7687694.f1.docx]

**Semi-structured focus group topic guide.**

______________________________________________________________________________

**Opening**

- Introduction
- Consent to record

**Topic 1: Understand the user experience of DPP participants wearing the CGM and receiving its output**

- What, if anything, did you like about wearing the CGM?
- What, if anything, did you dislike about wearing the CGM?
- Describe how you felt to be able to see your glucose levels continuously.
- What are some of the challenges that you experienced with wearing the CGM, if any?
  - Probe: How did you overcome them?

**Topic 2: Understand the user experience of DPP participants receiving the brief educational session and reading the handout**

- Please tell me ways in which we can improve the CGM education session for future participants.
  - Prompt: Were there any concepts that you found difficult to understand or confusing?
- Describe your experience in using the “Glucose Tracking” sheet.
  - Prompt: What were the benefits or burdens of tracking your food intake and corresponding glucose level?

**Topic 3: Identify the type of information that DPP participants need to use the CGM and relate CGM output with diet and physical activity behaviors**

- Describe how you used the data from CGM, if at all.
  - PROMPT: did anyone make changes to your diet in response to seeing your glucose levels?
    - Follow-up: Can you describe a specific change you made?
  - PROMPT: did anyone make changes to your physical activity in response to seeing your glucose levels?
    - Follow-up: Can you describe a specific change you made?
- Did you think you needed to wear CGM for more than 2 weeks to understand and benefit from the data or was this enough time? Explain.

**Topic 4: Identify potential barriers to making recommended behavioral changes and areas where additional support may be required**

- Describe any difficulties you experienced in making changes to your diet while wearing the CGM, if at all.
  - Probe: How did you overcome this difficulty?
- Describe any difficulties you experienced in making changes to your physical activity while wearing the CGM, if at all.
  - Probe: How did you overcome this difficulty?
- What additional support and resources, if any, do you wish you had while wearing the CGM?

**Closing**

- Is there anything else you’d like to share with us about the CGM or the education session, or anything else we talked about today?

___________________________________________________________________________
